# Dual modulation on glial cells by tetrahydroxystilbene glucoside protects against dopamine neuronal loss

**DOI:** 10.1186/s12974-018-1194-5

**Published:** 2018-05-25

**Authors:** Yanzhen Zhou, Guoqing Wang, Daidi Li, Yanying Wang, Qin Wu, Jingshan Shi, Feng Zhang

**Affiliations:** 1 Key Laboratory of Basic Pharmacology of Ministry of Education and Joint International Research Laboratory of Ethnomedicine of Ministry of Education, Zunyi Medical University, Zunyi, Guizhou China; 2 Department of Ear-Nose-Throat Surgery, The Affiliated Hospital of Zunyi Medical University, Zunyi, Guizhou China

**Keywords:** Parkinson’s disease, Neuroinflammation, Neurotrophic effects, Tetrahydroxystilbene glucoside, Neuroprotection

## Abstract

**Background:**

Microglia-mediated neuroinflammation is recognized to mainly contribute to the pathogenesis of Parkinson’s disease (PD). Tetrahydroxystilbene glucoside (TSG) has been proved to be beneficial for health with a great number of pharmacological properties. We examined the effects of TSG against dopamine (DA) neuronal loss towards development of a PD treatment strategy.

**Methods:**

Substantia nigral stereotaxic single injection of lipopolysaccharide (LPS)-induced rat DA neuronal damage was employed to investigate TSG-produced neuroprotection. In addition, primary rat midbrain neuron-glia co-cultures were performed to explore the underlying mechanisms.

**Results:**

Daily intraperitoneal injection of TSG for seven consecutive days significantly attenuated LPS-induced loss of DA neurons in the substantia nigra. In addition, glia-dependent mechanisms were responsible for TSG-mediated neuroprotection. First, TSG ameliorated microglia-mediated neuroinflammation and the subsequent production of various pro-inflammatory and neurotoxic factors. Second, astroglial neurotrophic factor neutralization weakened TSG-mediated neuroprotection, showing that TSG was protective in part via increasing astroglia-derived neurotrophic factor secretion.

**Conclusions:**

TSG protects DA neurons against LPS-induced neurotoxicity through dual modulation on glial cells by attenuating microglia-mediated neuroinflammation and enhancing astroglia-derived neurotrophic effects. These findings might open new alternative avenues for PD treatment.

## Background

Parkinson’s disease (PD) is among the most common and age-related neurodegenerative disease. It is characterized by slow and progressive loss of dopamine (DA) neurons in the midbrain substantia nigra (SN) and the consequent severe decrease of DA levels in the striatum. The pathological hallmark of PD is the formation of α-synuclein-containing Lewy body in DA neurons. Current drug treatments are mainly focused on symptomatic controls and long-term application results in a loss of drug efficacy and serious adverse effects and importantly could not halt the neurodegeneration.

Although mitochondrial dysfunction, oxidative stress, and environmental exposure have been identified to be closely associated with the pathogenesis of PD, the mechanisms underlying the progressive feature of DA neurodegeneration are not fully elucidated. Recently, neuroinflammation is increasingly implicated in the pathogenesis of neurodegenerative diseases, such as PD, Alzheimer’s disease (AD), and Huntington’s disease. The hallmark of neuroinflammation is the glial activation, particularly microglial activation [1]. Microglia, the resident immune cells in the central nervous system (CNS), serve critical roles in immune surveillance under normal conditions. Upon activation by brain damage or inflammogen, microglia release various kinds of pro-inflammatory and cytotoxic factors, such as cytokines, reactive oxygen species (ROS), and reactive nitrogen species (RNS). The accumulation of these factors contribute to the progressive loss of DA neurons. However, the continuing dying/dead DA neurons, in turn, result in the secondary activation of microglia and the activated microglia further induce DA neuronal damage [2]. Together, a vicious cycle leading to the prolonged neuroinflammation and the progressive DA neurodegeneration is created [3]. Thus, the inhibition of microglia-mediated neuroinflammation is becoming a promising therapeutic potential for PD treatment.

Growing evidence demonstrated that astroglia also play an important role in PD development and become the prime targets for PD treatment [4]. As the most abundant CNS cell type, astroglia serve the role of housekeeping works including the CNS development, neurotransmitters uptake, immune system regulation, and neurotrophic factor secretion [5]. For the endogenous substances, the neurotrophic factors, such as brain-derived neurotrophic factor (BDNF) and glial cell line-derived neurotrophic factor (GDNF), have emerged as the key factors in promoting the neuronal survival, growth, and differentiation [6]. The absence and depletion of these neurotrophic factors are revealed to be closely related to the pathogenesis of PD [7]. Therefore, strategies of the application of GDNF and BDNF, as well as the proper mimetic agents on an appropriate action site to replenish endogenous neurotrophic factors, might provide a critical neuronal support in PD.

Tetrahydroxystilbene glucoside (TSG) is well studied and holds a great number of pharmacological properties, such as free radical scavenging, anti-oxidant, and anti-inflammation effects [8]. Recent studies indicate TSG exerted neuroprotection against cerebral ischemia and neurodegenerative diseases [9]. However, the mechanisms underlying TSG-mediated neuroprotection require further illumination. In this study, lipopolysaccharide (LPS)-induced rat midbrain DA neuronal damage model was applied to investigate TSG-mediated neuroprotection. Primary rat midbrain neuron-glia co-cultures were performed as an in vitro model to explore the underlying mechanisms. Specially, these findings might open new alternative avenues for PD treatment.

## Methods

### Animals and treatment

Male Sprague Dawley (SD) rats (200–240 g) were obtained from the Experimental Animal Centre of the Third Military Medical University (Chongqing, China; Specific-pathogen free Grade II; Certificate No. scxk 2002003). All animal experiments were performed in accordance with the Chinese Guidelines of Animal Care and Welfare, and the present study was approved by the Animal Care and Use Committee of Zunyi Medical University (Zunyi, China). To explore the neuroprotective effects of TSG (the National Institute for the Control of Pharmaceutical and Biological Products, Beijing, China) on LPS-induced DA neurotoxicity, male rats were treated with a single intranigral injection of LPS (*Escherichia coli* strain O111:B4, Sigma-Aldrich, MO, USA; 5 μg) into SN pars compacta on one side of the rat brain followed by the coordinates: 4.8 mm posterior to bregma, 1.7 mm lateral to the midline, and 8.2 mm ventral to the surface of the skull [10]. TSG (10 and 50 mg/kg/day, intraperitoneal injection) was administrated for seven consecutive days after LPS injection. Control animals received equivolume injections of saline.

### Primary rat midbrain neuron-glia and neuron-astroglia cultures

Primary neuron-glia cultures were prepared from the ventral midbrain tissues of the embryonic day 14 ± 0.5 days of SD rats [11]. The whole brain was aseptically removed, and the mesencephalon was dissected. After the blood vessels and meninges were removed, the mesencephalic tissues were dissociated by the mechanical trituration and the dissociated cells were seeded at 5 × 10^5^/well and 1 × 10^5^/well in poly-d-lysine-coated 24- and 96-well plates, respectively. Seven-day-old cultures were used for drug treatments. At the time of treatment, immunocytochemical analysis indicated that the rat neuron-glia cultures consisted of 10% microglia, 50% astrocytes, 40% neurons, and 1% DA neurons. Primary midbrain neuron-astroglia cultures were obtained by suppressing microglia proliferation with leu-leu methyl ester (Sigma-Aldrich, MO, USA; 1.5 mM) added to neuron-glia cultures 1 day after seeding the cells [12]. Seven days after initial cell seeding, the cultures were performed for drug treatment and the percentage of microglia in the cultures was < 1%.

### Primary rat microglia- and astroglia-enriched cultures

Primary microglia- and astroglia-enriched cultures were prepared from the whole brains of 1-day-old rat pups. The brain tissues, devoid of meninges and blood vessels, were dissociated by a mild mechanical trituration. The isolated cells (5 × 10^7^) were seeded in 150 cm^2^ culture flasks [13]. After a confluent monolayer of glial cells was established, microglia were separated from astroglia via shaking the flasks and primary microglia-enriched cultures were 95–98% pure for microglia. The remaining astroglia were detached with trypsin-ethylenediaminetetraacetic acid (EDTA) and seeded in the culture medium. After three consecutive passages, the immunocytochemical analysis indicated that primary astroglia-enriched cultures consisted of > 98% astroglia [14].

### Midbrain neuron-enriched and reconstituted neuron-microglia cultures

Primary rat midbrain neuron-enriched cultures were obtained from inhibiting glial cells proliferation with cytosine β-d-arabinofuranoside (Sigma-Aldrich, MO, USA; 8 μM) added to primary midbrain neuron-glia cultures 24 h after seeding the cells. Seven days later, the neuron-enriched cultures consisted of 90% neurons, 10% astroglia, and < 0.1% microglia. Primary neuron-microglia cultures were obtained by adding primary microglia (5 × 10^4^/well) from primary microglia-enriched cultures back to neuron-enriched cultures [15].

### [^3^H] DA uptake assay

Primary midbrain neuron-glia cultures were incubated with [^3^H]DA (PerkinElmer Life Sciences Inc., Boston, MA, USA) in Krebs-Ringer buffer at 37 °C for 20 min. Liquid scintillation counting was applied to detect the radioactivity. Mazindol was used to block the non-specific DA uptake. After the cultures were washed for three times with ice-cold Krebs–Ringer buffer and lysed with sodium hydroxide (NaOH), the lysate was mixed with scintillation fluid and the radioactivity was detected through the liquid scintillation counter. The specific [^3^H] DA uptake was calculated by subtracting the radioactivity amount obtained in the presence of mazindol from that obtained in the absence of mazindol [11].

### Immunocytochemical staining

DA neurons were recognized with an anti-tyrosine hydroxylase (TH) antibody (Sigma-Aldrich, MO, USA). Microglia activation was detected with an anti-CR3 complement receptor (OX-42) antibody (Pharmingen, CA, USA). Astroglia were immunostained with an anti-glial fibrillary acidic protein (GFAP) antibody (Abacm, Cambridge, UK). For morphological analysis, the images were recorded by a charge-coupled device camera and analyzed by the MetaMorph software. For visual counting of TH-positive neurons, four representative areas per well in the 24-well plate were counted. In each condition, three wells were used for cell counting.

### Nitric oxide (NO), tumor necrosis factor-alpha (TNFα), interleukin-1 beta (IL-1β), and prostaglandin-E_2_ (PGE_2_) assay

NO was accessed by measuring the accumulated levels of nitrite in the culture medium with the Griess reagent. TNFα, IL-1β, and PGE_2_ levels in the culture supernatants were measured with enzyme-linked immunosorbent assay (ELISA) kits from R&D Systems (Minneapolis, MN, USA).

### BDNF and GDNF measurement by ELISA

BDNF and GDNF production in midbrain neuron-glia culture medium were quantified with ELISA kits according to the procedures provided by the manufacturer from Promega (Madison, WI, USA).

### Superoxide assay

The production of superoxide was detected by measuring the superoxide dismutase (SOD)-inhibitable reduction of the water-soluble tetrazolium salt (WST-1) (Dojindo Laboratories, MD, USA). Primary microglia-enriched cultures seeded in 96-well plate were washed twice with Hank’s balanced salt solution (HBSS) without phenol red. Then, cells were incubated at 37 °C for 30 min with control or TSG in HBSS (50 μl/well). Subsequently, HBSS (50 μl) with and without SOD (50 U/ml) was added to each well along with WST-1 (1 mM, 50 μl) in HBSS, and 50 μl of control or LPS. The absorbance at 450 nm was read via a SpectraMax Plus microplate spectrophotometer in every 5 min till 1 h. The different absorbance observed in the presence and absence of SOD was considered to be the amount of superoxide production.

### Intracellular ROS assay

Intracellular ROS were determined by the fluorescence probe dichlorodihydrofluorescein diacetate (DCFH-DA, from Calbiochem, CA, USA) assay. Primary microglia-enriched cultures were seeded in 96-well plate and then exposed to DCFH-DA for 1 h, followed by TSG treatment for 30 min and then stimulation with HBSS containing LPS. After incubation at 37 °C for 30 min, the fluorescence was read at 485 nm for excitation and 530 nm for emission through a SpectraMax Gemini XS fluorescence microplate reader.

### Western blotting

For the subcellular fractions, primary microglia-enriched cultures were lysed in hypotonic lysis buffer and incubated on ice for 30 min, and then subjected to homogenization. The lysates were loaded onto a sucrose gradient in lysis buffer, and the supernatant above the sucrose gradient was performed as the cytosolic fraction. The pellet was solubilized in hypotonic lysis buffer and was applied as the membranous fraction. For the whole cell lysate extraction, cultures were washed with cold phosphate-buffered saline (PBS) and lysed with cell lysis buffer. The lysates were incubated on ice for 30 min and then centrifuged at 12,000×*g* for 25 min. Protein levels were quantified via bicinchoninic acid (BCA) assay. Membranes were blocked with 5% non-fat milk and then incubated with the following primary antibodies: β-actin, TH, ionized calcium-binding adapter molecule-1 (Iba-1), α-synuclein, p47, p67, gp91, BDNF, GDNF (Abcam, Cambridge, UK), phospho-p65 (p-p65), p65, phospho-IKK (p-IKK), anti-IKK, phospho-p38 (p-p38), p38, phospho-JNK (p-JNK), JNK (Cell Signaling Technology, MA, USA), and horseradish peroxidase-conjugated secondary antibodies (Vector Laboratories, CA, USA). All the antibodies were diluted between 1:200 and 1:1000. The blots were developed with the enhanced ECL reagent.

### Immunohistochemistry and cell counting in substantia nigra

Rat brains were cut on a horizontal sliding microtome into 35 μm transverse free-floating sections. A total of 36 consecutive brain slices throughout the entire SN were harvested, and every sixth section was processed for the immunocytochemical staining. Digital images of TH-positive DA neurons, OX-42-positive microglia, and GFAP-positive astroglia in the SN were obtained on an Olympus microscope using an attached Polaroid digital microscope camera (Polaroid®, Cambridge, MA, USA). Quantification of DA neurons was performed through visually counting the number of TH-positive neuronal cell bodies blindly by two investigators, and the results were analyzed from the average. The mean value for SN TH-positive neuronal numbers was then deduced by averaging the counts of six sections for each animal. Densitometric analysis using AlphaImager software package was performed.

### Rotarod test

Rotarod test was performed for the study of muscular coordination. It contained cylindrical arrangement of thin steel rods, which was divided into two parts by compartmentalization to test two rats at same time. In the train, the speed was set at 10 cycles per min and cut-off time was 180 s. Prior to the start of the test, rats were trained on rotarod until they remained on the rod at least for the cut-off time. Rats were allowed to remain stationary for a while at 0 rpm. The rotational speed was steadily increased to 10 rpm in 20 s interval till rats fell off the rungs. Rat behavior changes were tested for two investigators, and the mean duration time remained on the rod was recorded [16].

### HPLC analysis of striatal neurotransmitter levels

The striatal levels of DA and its metabolites 3,4-dihydroxyphenylacetic acid (DOPAC) and homovanillic acid (HVA) were determined by HPLC coupled with electrochemical detection. Briefly, striatal tissues were sonicated in perchloric acid (PCA) (20% wt/vol) containing the internal standard 3, 4-dihydroxybenzylamine (10 mg wet tissue/ml). The homogenate was centrifuged, and an aliquot of the supernatant was injected into HPLC equipped with a C_18_ column (Dionex, Germering, Germany). The mobile phase was comprised of acetonitrile, tetrahydrofuran, and monochloroacetic acid (pH 3.0) containing of EDTA (50 mg/L) and sodium octyl sulfate (200 mg/L). The amount of DA, DOPAC, and HVA were measured by comparison of peak height ratio of tissue sample with standards and expressed in the quality of wet weight of tissue [12].

### Statistical analysis

Data were expressed as mean ± standard error of the mean (SEM). Statistical significance was analyzed by two-way ANOVA using GraphPad Prism software (GraphPad Software Inc., San Diego, CA, USA). When ANOVA indicated the significant differences, pairwise comparisons between means were accessed by Bonferroni’s post hoc *t* test with correction. A value of *P* < 0.05 was considered statistically significant.

## Results

### TSG produces neuroprotection against LPS-induced DA neurotoxicity

**Fig. 1:**
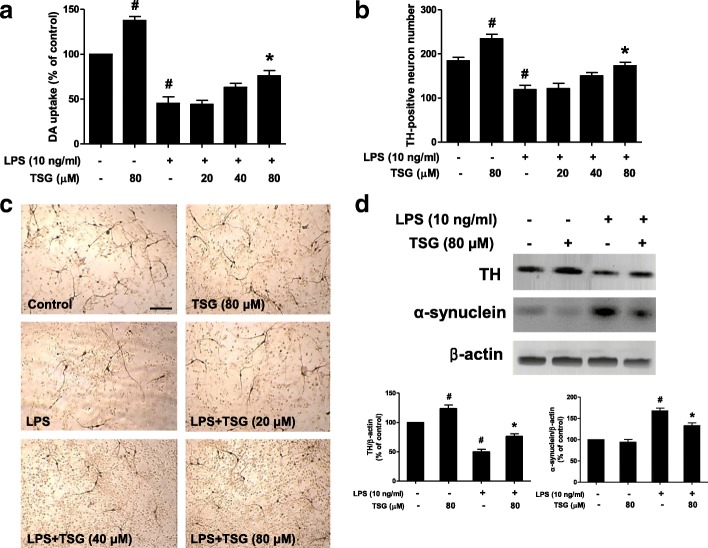
TSG produced neuroprotection against LPS-induced DA neurotoxicity. Rat primary midbrain neuron-glia cultures were treated with TSG (20–80 μM) for 30 min before LPS (10 ng/ml) application. Seven days later, LPS-elicited DA neurotoxicity was quantified by [^3^H] DA uptake assay (**a**), the two-way ANOVA interactions analysis between LPS and TSG treatments showed *F* = 0.48 and *P* = 0.508) and TH-positive neuron counting through the immunocytochemical analysis (**b**), the two-way ANOVA interactions analysis showed *F* = 0.036 and *P* = 0.853). Representative images of immunostaining from three experiments were indicated (**c**). Scale bar = 200 μm. The protein levels of TH and α-synuclein were determined by western blotting. The ratio of densitometry values of TH and α-synuclein with β-actin was assessed and normalized to each respective control group (**d**), TH: the two-way ANOVA interactions analysis of showed *F* = 0.097 and *P* = 0.764; α-synuclein: the two-way ANOVA interactions analysis of showed *F* = 1.602 and *P* = 0.232). Data were the mean ± SEM from three independent experiments performed in triplicate. ^#^*P* < 0.05 compared with control cultures; *P < 0.05 compared with LPS-treated cultures

Rat primary midbrain neuron-glia cultures were treated with TSG (20–80 μM) for 30 min before LPS (10 ng/ml) application. Seven days later, the LPS-elicited DA neurotoxicity was quantified by [^3^H] DA uptake assay and TH (a marker of DA neuron)-positive neuronal counting. As shown in Fig. 1a, [^3^H] DA uptake assay indicated that LPS decreased the capacity of the cultures to take up DA by approximately 55% compared with the control cultures and TSG attenuated this reduction in a concentration-dependent manner. Moreover, TSG alone increased DA capacity of up to 140% that of control. Subsequently, the neuroprotective effects of TSG on LPS-induced neurotoxicity were further confirmed by TH-positive neurons counting. As shown in Fig. 1b, c, consistent with the results from [^3^H] DA uptake assay, TSG ameliorated LPS-induced DA neuron number reduction. Also, TSG alone increased the TH-positive neuronal number up to 125% that of control. To further confirm TSG-mediated neuroprotection, the effects of TSG on LPS-induced TH and α-synuclein protein expressions were determined by western blotting. Compared with the control cultures, TSG alone increased TH protein expression. Compared with LPS-treated cultures, TSG attenuated LPS-induced decrease of TH protein expression and increase of α-synuclein protein level as shown in Fig. 1d.

### Microglia and astroglia are indispensable for TSG neuroprotection

**Fig. 2:**
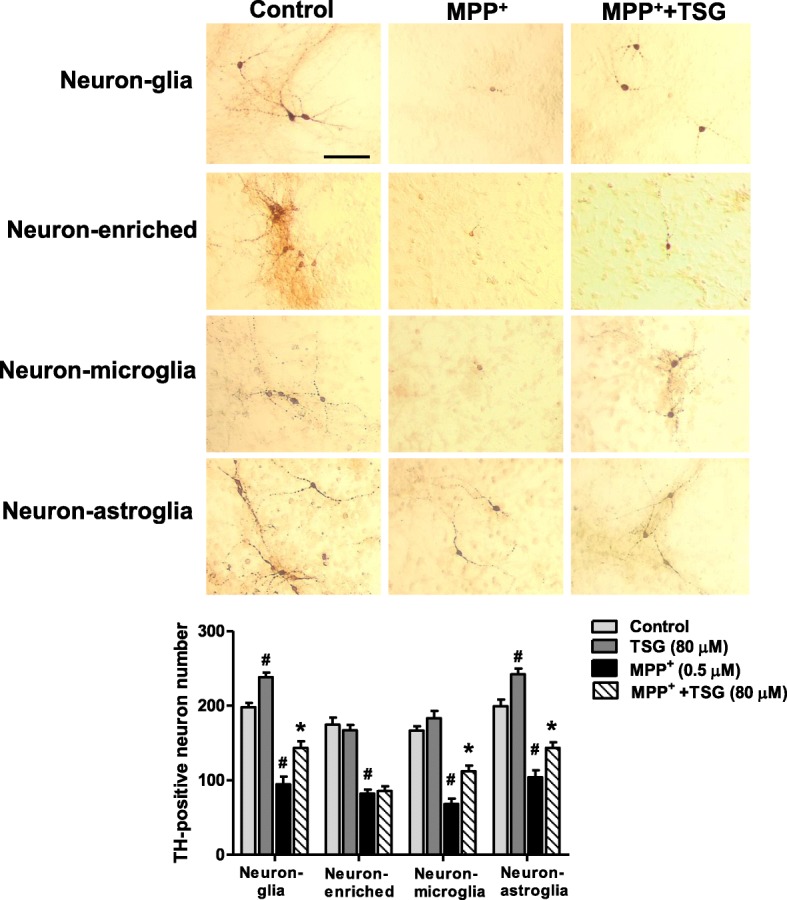
Microglia and astroglia were indispensable for TSG neuroprotection. Four types of primary cultures were treated with TSG (80 μM) for 30 min followed by MPP^+^ (0.5 μM) addition. Seven days later, the DA neurotoxicity was measured by TH-positive neuron counting. Data were the mean ± SEM from three independent experiments performed in triplicate (Neuron-glia cultures: the two-way ANOVA interactions analysis of showed *F* = 0.270 and *P* = 0.617; Neuron-enriched cultures: the two-way ANOVA interactions analysis of showed *F* = 0.610 and *P* = 0.457; Neuron-microglia cultures: the two-way ANOVA interactions analysis of showed *F* = 3.043 and *P* = 0.119; Neuron-astroglia cultures: the two-way ANOVA interactions analysis of showed *F* = 0.033 and *P* = 0.861). Scale bar = 200 μm. ^#^*P* < 0.05 compared with control cultures; *P < 0.05 compared with MPP^+^-treated cultures

To determine which cell type was involved in TSG-mediated neuroprotection, four types of primary cultures including midbrain neuron-glia, neuron-microglia, neuron-astroglia, and neuron-enriched cultures were prepared. These cultures were treated with TSG (80 μM) 30 min prior to 1-methyl-4-phenylpyridinium (MPP^+^, Sigma-Aldrich, MO, USA; 0.5 μM) addition. MPP^+^ was the active metabolite of 1-methyl-4-phenyl-1,2,3,6-tetrahydropyridine (MPTP) and directly caused DA neuronal death. Neurotoxic factors such as α-synuclein released by the dying/dead DA neurons in turn led to microglial activation and further produced DA neurotoxicity [17]. In these four culture systems shown in Fig. 2, MPP^+^ significantly decreased DA neuronal number and TSG differentially improved MPP^+^-elicited DA neurotoxicity in neuron-glia, neuron-microglia, and neuron-astroglia except for neuron-enriched cultures. These results demonstrated that TSG-mediated neuroprotection on DA neurons was glia-dependent. Additionally, TSG alone increased DA neuronal number in neuron-glia and neuron-astroglia cultures but not in either neuron-enriched or neuron-microglia cultures, suggesting astroglia participated in TSG-mediated neurotrophic effects.

### TSG inhibits microglia-mediated neuroinflammation

**Fig. 3:**
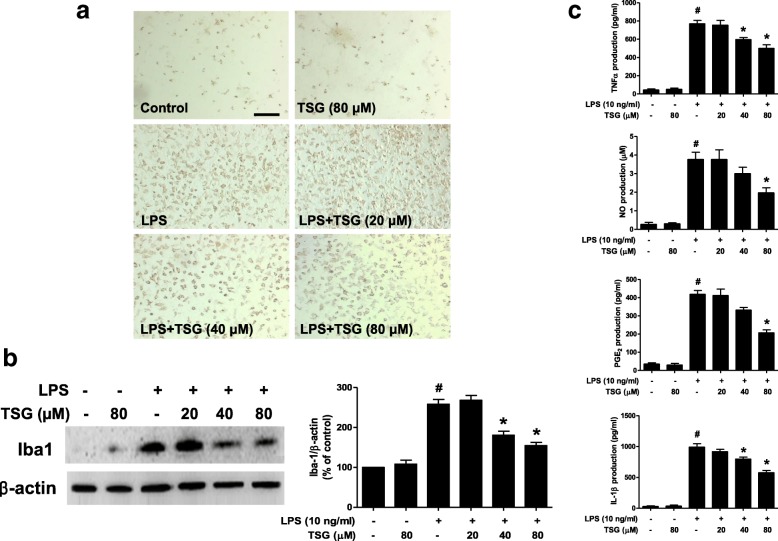
TSG inhibited microglia-mediated neuroinflammation. Rat primary midbrain neuron-glia cultures were treated with TSG (20–80 μM) for 30 min before LPS (10 ng/ml) stimulation. Seven days later, LPS-induced microglial activation was analyzed by immunostaining with an anti-OX-42 antibody (**a**) and Iba-1 protein expression by western blotting (**b**); the two-way ANOVA interactions analysis showed *F* = 3.963 and *P* = 0.127). Images presented were representative of three independent experiments. Scale bar = 200 μm. Also, the total cell protein was harvested and the Iba-1 protein expression was quantified. The ratio of densitometry values of Iba-1 and β-actin was assessed and normalized to the control group. In addition, rat primary midbrain neuron-glia cultures were treated with TSG (20–80 μM) for 30 min and then stimulated by LPS (10 ng/ml). After LPS treatment for 6 and 24 h, an aliquot of the culture medium was harvested for ELISA analysis of TNFα, IL-1β, and PGE_2_ and Griess reaction analysis of nitrite levels (**c**). TNFα: the two-way ANOVA interactions analysis of showed *F* = 3.098 and *P* = 0.112; NO: the two-way ANOVA interactions analysis of showed *F* = 4.005 and *P* = 0.091; PGE_2_: the two-way ANOVA interactions analysis of showed *F* = 4.453 and *P* = 0.087; IL-1β: the two-way ANOVA interactions analysis of showed *F* = 4.786 and *P* = 0.063). Data the mean ± SEM from three independent experiments performed in triplicate. ^#^*P* < 0.05 compared with control cultures; ^*^P < 0.05 compared with LPS-treated cultures

Rat primary midbrain neuron-glia cultures were treated with TSG (20–80 μM) for 30 min before LPS (10 ng/ml) stimulation. Seven days later, the LPS-induced microglial activation was analyzed by immunostaining with an anti-OX-42 (a marker of activated microglia used for immunostaining) antibody. As shown in Fig. 3a, microglia in LPS-treated cultures exhibited an enlarged cell body and irregular shapes from resting round and small cells to the highly activated amoeboid status, which was in parallel with the loss of TH-positive neurons. TSG significantly attenuated LPS-induced microglial activation. To further explore the inhibitory properties of TSG on microglial activation, the whole cell lysis was harvested and the effects of TSG on the protein expression of Iba-1 (a marker of microglia used for western blotting) were investigated. As shown in Fig. 3b, TSG decreased LPS-induced Iba-1 protein expression.

The effects of TSG on the production of LPS-induced pro-inflammatory factors were further determined. As the peak levels of pro-inflammatory factors released from microglia varied in terms of time and quantities, different time points were adjusted for the measurement of each pro-inflammatory factor: TNFα at 6 h and IL-1β, PGE_2_, and NO at 24 h after LPS treatment. As shown in Fig. 3c, TSG significantly reduced LPS-induced production of TNFα, IL-1β, PGE_2_, and NO in the neuron-glia cultures medium.

**Fig. 4:**
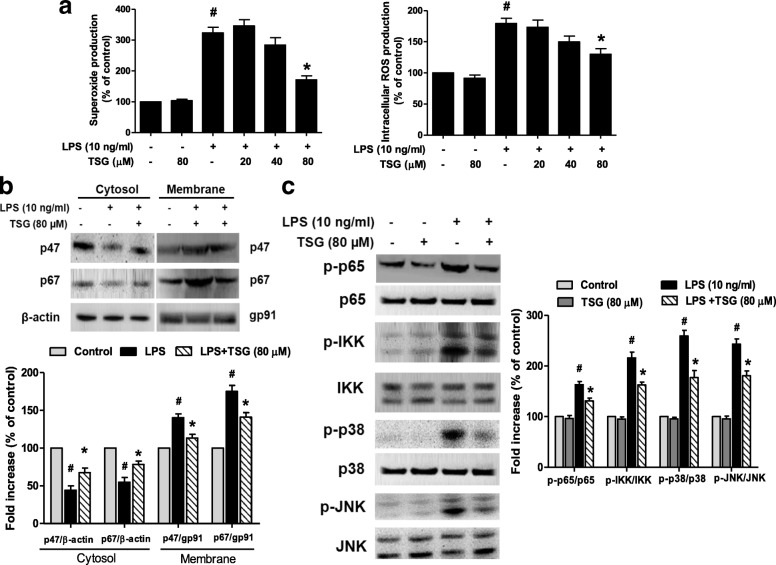
TSG suppressed microglial ROS production and NADPH oxidase signaling pathway activation. Primary microglia-enriched cultures were treated with TSG for 30 min before LPS stimulation for 15 min. The extracellular superoxide production was detected by SOD-inhibitable reduction of WST-1 and the intracellular ROS level was determined with DCFH-DA assay (**a**), Superoxide: the two-way ANOVA interactions analysis of showed *F* = 4.076 and *P* = 0.089; intracellular ROS: the two-way ANOVA interactions analysis of showed *F* = 2.451 and *P* = 0.137). The subcellular fractions were isolated to perform western blotting for p47 and p67 levels in membrane and cytosolic fractions of microglia. β-actin and gp91 were applied as internal cytosolic and membrane controls, respectively (**b**), Cytosol-p47: the two-way ANOVA interactions analysis of showed *F* = 2.273 and *P* = 0.206; Cytosol-p67: the two-way ANOVA interactions analysis of showed *F* = 3.798 and *P* = 0.109; Membrane-p47: the two-way ANOVA interactions analysis of showed *F* = 1.367 and *P* = 0.283; Membrane-p67: the two-way ANOVA interactions analysis of showed *F* = 4.084 and *P* = 0.091). The whole cell levels of microglial phosphorylated p65 (p-p65), IKK (p-IKK), p38 (p-p38) and JNK (p-JNK) compared to total p65, IKK, p38 and JNK were investigated by western blotting (**c**), p65: the two-way ANOVA interactions analysis of showed *F* = 1.425 and *P* = 0.279; IKK: the two-way ANOVA interactions analysis of showed *F* = 4.615 and *P* = 0.061; p38: the two-way ANOVA interactions analysis of showed *F* = 2.784 and *P* = 0.114; JNK: the two-way ANOVA interactions analysis of showed *F* = 4.467 and *P* = 0.066). Data were expressed as a percentage of the control cultures and were the mean ± SEM from 3 independent experiments performed in triplicate. ^#^*P* < 0.05 compared with control cultures; ^*^P < 0.05 compared with LPS-treated cultures

To detect whether TSG was capable to decrease LPS-induced ROS production, primary microglia-enriched cultures were treated with TSG for 30 min before LPS stimulation for 15 min. As shown in Fig. 4a, LPS significantly led to the increased production of extracellular superoxide and intracellular ROS and these increases could be attenuated by TSG pretreatment. Since NADPH oxidase is the key enzyme required for the production of superoxide and intracellular ROS in activated immune cells, the effects of TSG on NADPH oxidase signaling activation were next explored. Western blotting demonstrated that LPS significantly induced the translocation of NADPH oxidase subunit p47 and p67 from cytosol to membrane and TSG could ameliorate LPS-induced the increase of p47 and p67 translocation shown in Fig. 4b.

Additionally, it is well known that MAPK and NF-κB pathways were involved in the regulation of immune responses. Therefore, the effects of TSG on LPS-induced MAPK and NF-κB signaling pathway activation were investigated. Primary microglia-enriched cultures were treated with TSG for 30 min and then stimulated with LPS for 15 min. As shown in Fig. 4c, TSG attenuated LPS-induced phosphorylation of p38, JNK, p65, and IKK in microglia.

### Astroglial neurotrophic factors participate in TSG neuroprotection

**Fig. 5:**
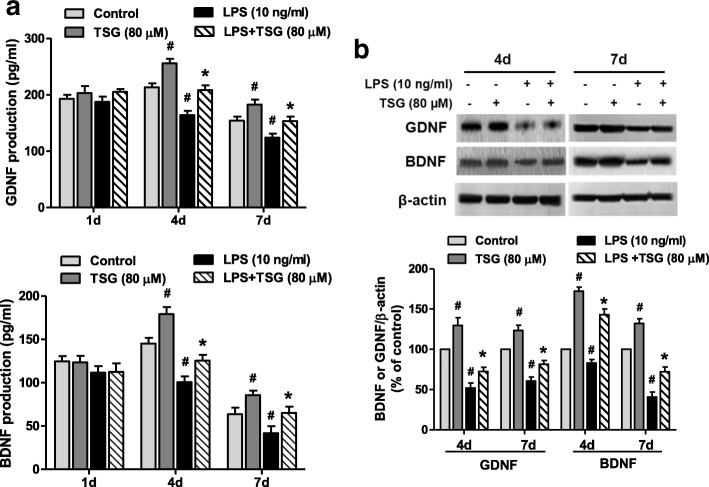
TSG enhanced neurotrophic factors release in LPS-induced DA neurotoxicity. Primary neuron-glia cultures were treated with TSG for 30 min followed by LPS addition. The levels of GDNF and BDNF in neuron-glia culture supernatant 1, 4, and 7 days after TSG treatment were measured by ELISA, respectively (**a**) (GDNF-1 day: the two-way ANOVA interactions analysis of showed *F* = 0.169 and *P* = 0.692; GDNF-4 day: the two-way ANOVA interactions analysis of showed *F* = 0.012 and *P* = 0.917; GDNF-7 day: the two-way ANOVA interactions analysis of showed *F* = 0.004 and *P* = 0.951; BDNF-1 day: the two-way ANOVA interactions analysis of showed *F* = 0.023 and *P* = 0.884; BDNF-4 day: the two-way ANOVA interactions analysis of showed *F* = 0.393 and *P* = 0.548; BDNF-7 day: the two-way ANOVA interactions analysis of showed *F* = 0.009 and *P* = 0.928). The protein levels of BDNF and GDNF in neuron-glia co-culture cells 4 and 7 days after TSG treatment were determined by western blotting. Quantified results were shown as a percentage of the control cultures (**b**) (GDNF-4 day: the two-way ANOVA interactions analysis of showed *F* = 0.518 and *P* = 0.492; GDNF-7 day: the two-way ANOVA interactions analysis of showed *F* = 0.100 and *P* = 0.760; BDNF-4 day: the two-way ANOVA interactions analysis of showed *F* = 1.594 and *P* = 0.242; BDNF-7 day: the two-way ANOVA interactions analysis of showed *F* = 0.004 and *P* = 0.952). Data were the mean ± SEM from three independent experiments performed in triplicate. ^#^*P* < 0.05 compared with control cultures; ^*^P < 0.05 compared with LPS-treated cultures

Neuron-glia cultures were treated with TSG for 30 min followed by LPS addition. One, 4, and 7 days after TSG treatment, the production of GDNF and BDNF in the culture supernatant were measured by ELISA, respectively. As shown in Fig. 5a, no significant difference of GDNF or BDNF production among the control, TSG alone, LPS, and LPS + TSG treatment groups was indicated 1 day after TSG treatment. However, from TSG treatment for 4 days, TSG alone increased BDNF and GDNF levels compared with control cultures and TSG also attenuated LPS-decreased BDNF and GDNF secretion in the culture medium. Next, the potential effects of TSG on BDNF and GDNF protein expressions were explored. As shown in Fig. 5b, similar to BDNF and GDNF release, TSG alone induced BDNF and GDNF protein expressions and TSG ameliorated LPS-reduced BDNF and GDNF protein levels from TSG treatment for 4 days.

**Fig. 6:**
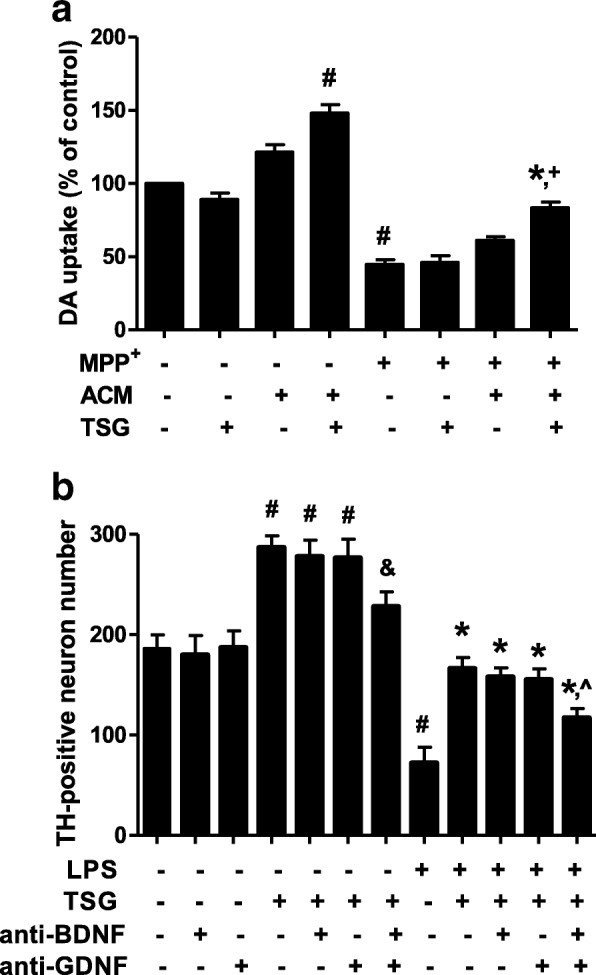
Astroglial neurotrophic factors participated in TSG neuroprotection. **a** The astroglia-conditioned medium (ACM) was first prepared from primary astroglia-enriched cultures. Primary astroglia were treated with TSG (80 μM) and incubated for 2 days and then astroglia-conditioned medium with TSG treatment (ACM-TSG) and the ACM without any treatment were harvested and dialyzed and added back to primary neuron-enriched cultures followed by the incubation for 7 days. DA neuronal function was evaluated by [^3^H] DA uptake assay (The two-way ANOVA interactions analysis showed *F* = 4.987 and *P* = 0.052). ^#^*P* < 0.05 compared with control cultures; ^*^P < 0.05 compared with MPP^+^-treated cultures; ^+^*P* < 0.05 compared with MPP^+^ treated by ACM cultures. **b** Primary midbrain neuron-glia cultures were treated with TSG and then stimulated by LPS followed by the single treatment of anti-BDNF (20 μg/ml) or anti-GDNF antibodies (20 μg/ml) or combined these two antibodies. Seven days later, TH-positive neuronal counting analysis was performed to determine the role of astroglia-derived neurotrophic factors in TSG-mediated neuroprotection (The two-way ANOVA interactions analysis showed *F* = 4.548 and *P* = 0.081). Data were the mean ± SEM from three independent experiments performed in triplicate. ^#^*P* < 0.05 compared with control cultures; ^*^P < 0.05 compared with LPS-treated cultures; ^&^*P* < 0.05 compared with TSG alone-treated cultures; ^^^*P* < 0.05 compared with TSG plus LPS-treated cultures

To elucidate the role of astroglia in TSG-mediated neuroprotection, the astroglia-conditioned medium (ACM) was first prepared from primary astroglia-enriched cultures. Primary astroglia were treated with TSG (80 μM) and incubated for 2 days and then astroglia-conditioned medium with TSG treatment (ACM-TSG) and the ACM without any treatment were collected and dialyzed and added back to primary neuron-enriched cultures. Seven days later, [^3^H] DA uptake assay showed that compared with the control group in the neuron-enriched cultures, ACM-TSG increased DA uptake capacity, whereas TSG alone had no neurotrophic effects on DA neuron uptake. In addition, compared with MPP^+^-treated cultures, no significant neuroprotection was discerned in TSG-treated neuron-enriched cultures but ACM-TSG attenuated MPP^+^-induced decrease of DA uptake capacity shown in Fig. 6a.

To further explore astroglia-derived neurotrophic factors involved in TSG-mediated neuroprotection, primary midbrain neuron-glia cultures were treated with TSG for 30 min and then stimulated by LPS followed by the treatment of anti-BDNF or anti-GDNF antibodies or combined these two antibodies. Seven days later, TH-positive neuronal counting analysis indicated that compared with control cultures, TSG increased DA neuron number and this increase was reduced by anti-BDNF combined with anti-GDNF antibodies treatment. Compared with LPS-treated cultures, TSG ameliorated LPS-induced DA neuron number decrease and TSG-mediated neuroprotection was weakened by anti-BDNF together with anti-GDNF antibodies’ treatment shown in Fig. 6b.

### TSG attenuates LPS-induced SN DA neuronal loss in vivo

**Fig. 7:**
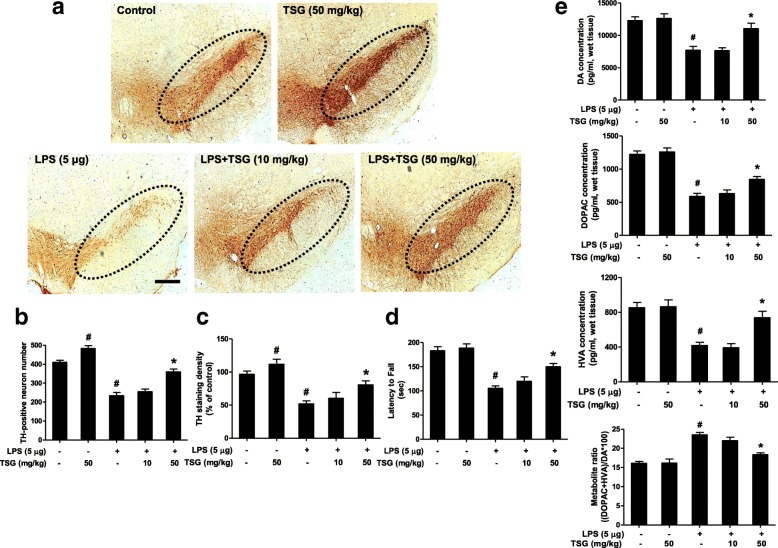
TSG attenuated LPS-induced SN DA neuronal loss in vivo. Rats were treated with a single intranigral injection of LPS into the SN on one side of rat brain followed by daily intraperitoneal injection of TSG for 7 days. The brain sections were immunostained with anti-TH antibody. The “ellipse” presented the area of SN (**a**). Scale bar = 200 μm. The TH-positive neuronal number in the SN was counted (**b**) ( the two-way ANOVA interactions analysis showed *F* = 3.200 and *P* = 0.111). The densitometry analysis of TH-positive DA neuronal staining from six evenly spaced brain sections from each rat was performed via ImageJ software (**c**) (the two-way ANOVA interactions analysis showed *F* = 1.309 and *P* = 0.286). Rat behavior changes were analyzed via rotarod test. The time remained on the rod was recorded (**d**) (the two-way ANOVA interactions analysis showed *F* = 4.119 and *P* = 0.089). The levels of DA and its metabolites, DOPAC, and HVA, in rat brain striatum, were measured by HPLC coupled with electrochemical detection (**e**) (DA: the two-way ANOVA interactions analysis showed *F* = 4.585 and *P* = 0.065; DOPAC: the two-way ANOVA interactions analysis showed *F* = 4.953 and *P* = 0.057; HVA: the two-way ANOVA interactions analysis showed *F* = 3.643 and *P* = 0.121; Metabolite ratio: the two-way ANOVA interactions analysis showed *F* = 3.934 and *P* = 0.108). Data were the mean ± SEM from six rats. ^#^*P* < 0.05 compared with control group; *P < 0.05 compared with LPS-treated group

Upon TSG-mediated neuroprotection in vitro demonstrated, TSG-protected DA neurons against LPS-induced neurotoxicity in vivo was further studied. Rats were treated with a single intranigral injection of LPS (5 μg in 2 μl of saline) into the SN on one side of the rat brain followed by daily intraperitoneal injection of TSG (10 and 50 mg/kg) treatment for 7 days. One day after the last TSG treatment, rat brains were sectioned and performed for DA neuron quantification by immunostaining analysis with an anti-TH antibody. As the results from DA neuronal quantification and densitometric analysis are shown in Fig. 7a–c, LPS decreased DA neuron number by approximately 50% compared with the control group and TSG could ameliorate this decrease. In addition, the DA neuronal number in TSG alone treatment group was increased up to 150% that of the control group. In rotarod test (Fig. 7d), TSG attenuated LPS-induced decrease in the time remained on the rod.

Besides DA neuronal number and rat behavior changes, the neuroprotective effects of TSG against LPS-induced depletion of striatal DA and its metabolites 3, 4-dihydroxyphenylacetic acid (DOPAC) and homovanillic acid (HVA) levels were next investigated by HPLC coupled with electrochemical detection. As shown in Fig. 7e, in parallel with the reduction of DA neuron number, a corresponding decrease in the content of DA, DOPAC, and HVA was indicated in LPS-treated group. TSG treatment led to various degrees of recovery of DA and its metabolites levels in the striatum. Moreover, the lower metabolite ratio [(HVA + DOPAC) × 100/DA] in TSG treatment group showed a decreased DA turnover compared with LPS treatment group.

**Fig. 8:**
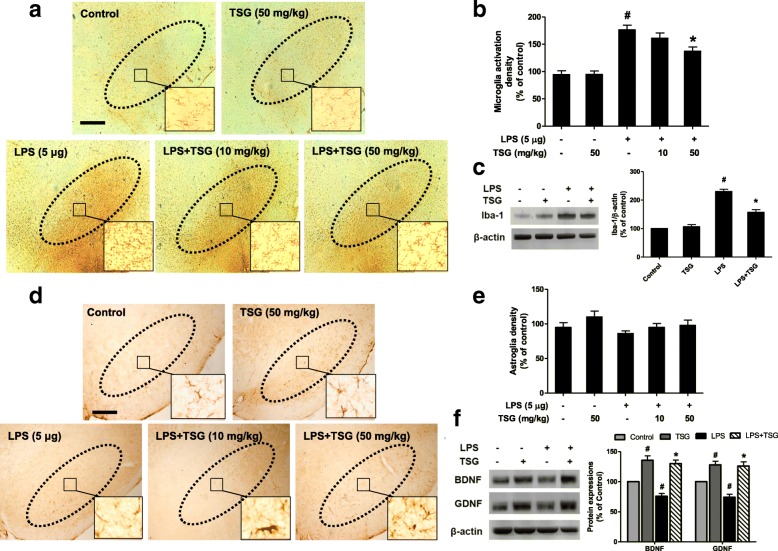
TSG modulated glial cells in vivo. Rat brains were sectioned and immunostained with an anti-OX-42 (**a**) and GFAP (**d**) antibodies. The images were representative of six rats in each group. Scale bar = 200 μm. The “ellipse” presented the area of SN. The densitometry analysis of SN OX-42-positive microglia (**b**) (the two-way ANOVA interactions analysis showed *F* = 4.318 and *P* = 0.072) and GFAP-positive astroglia (**e**) (the two-way ANOVA interactions analysis showed *F* = 0.059 and *P* = 0.815) from six evenly spaced brain sections from each rat was performed via ImageJ software. In addition, rat midbrains were collected to detect the protein expression of Iba-1 (**c**) (the two-way ANOVA interactions analysis showed *F* = 0.049 and *P* = 0.821) and BDNF and GDNF (**f**) (BDNF: the two-way ANOVA interactions analysis showed *F* = 2.928 and *P* = 0.113; GDNF: the two-way ANOVA interactions analysis showed *F* = 4.252 and *P* = 0.079) via western blotting. The ratio of densitometry values of Iba-1, BDNF, GDNF, and β-actin was assessed and normalized to control group. Data were expressed as a percentage of the control group and were the mean ± SEM from six rats. ^*#*^*P* < 0.05 compared with control group; *P < 0.05 compared with LPS-treated group

To further substantiate TSG-mediated DA neuroprotection through the inhibition of neuroinflammation, the effects of TSG on microglial activation in vivo were thus detected. Seven days after TSG treatment, rat brain sections were immunostained by an anti-OX-42 antibody. As shown in Fig. 8a, microglia in the control group exhibited a resting status. Upon activation by LPS injection, microglia were characterized by the larger size and thicker processes. Results from densitometric analysis and Iba-1 protein expression detection demonstrated that TSG treatment significantly attenuated LPS-elicited microglial activation shown in Fig. 8b, c. The in vivo results were in agreement with the findings from the in vitro studies. However, in astroglia immunostaining analysis with anti-GFAP (a marker of astroglia) antibody shown in Fig. 8d, e, TSG seemed not to have significant effects on astroglial activation. Additionally, data from protein expressions of neurotrophic factors in the midbrain indicated that TSG increased BDNF and GDNF protein expressions after LPS treatment shown in Fig. 8f.

## Discussion

**Fig. 9:**
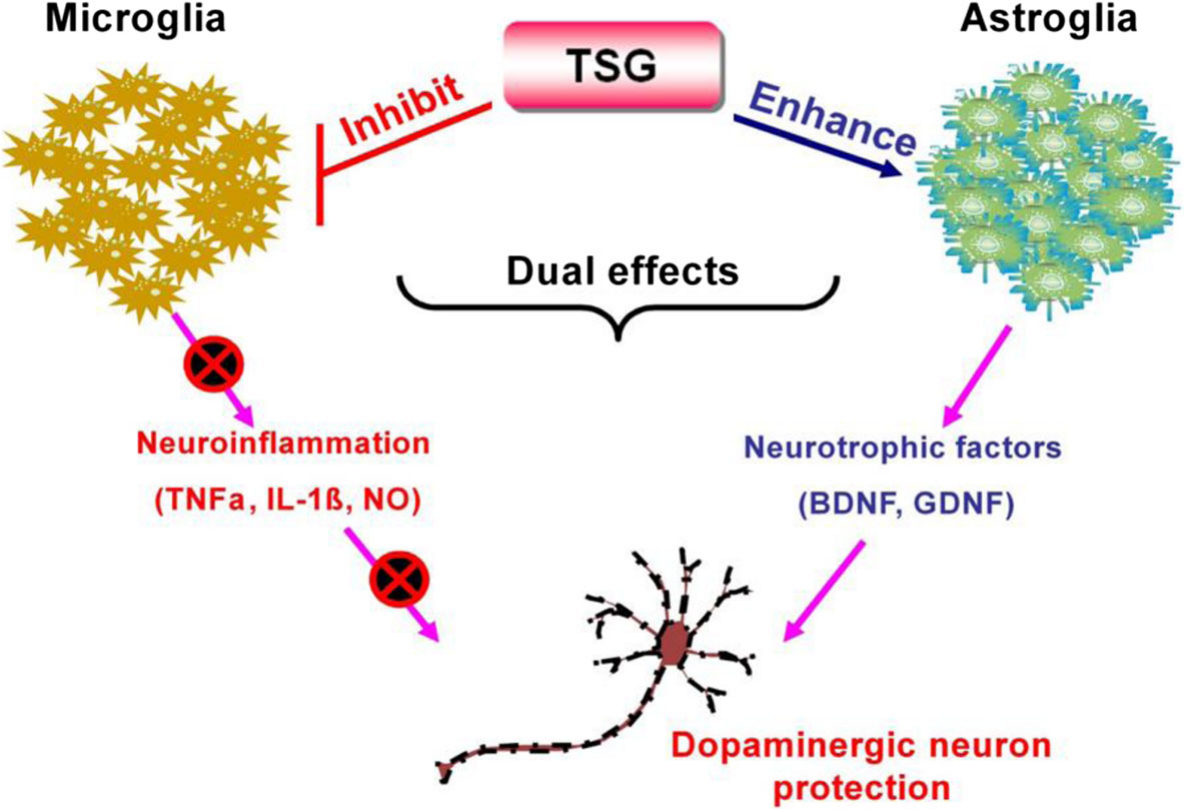
TSG produced neuroprotection against DA neuronal loss via the dual modulation on glial cells

The present study investigated the neuroprotective properties of TSG on LPS-induced DA neuronal damage to identify the therapeutic potential for PD. In vivo studies showed TSG significantly attenuated LPS-induced loss of SN DA neurons. In addition, TSG also produced neuroprotection against LPS-elicited DA neuronal damage in primary midbrain neuron-glia co-cultures. In vitro mechanistic studies revealed glia-dependent actions were responsible for TSG-mediated neuroprotection. First, TSG ameliorated microglia-mediated neuroinflammation and the subsequent production of various pro-inflammatory and neurotoxic factors. Second, astroglia-derived neurotrophic factors participated in TSG-mediated neuroprotection. Taken together, this study demonstrated TSG produced DA neuroprotection against LPS-induced neurotoxicity through dual modulation on glial cells by attenuating microglia-mediated neuroinflammation and enhancing astroglia-derived neurotrophic effects (Fig. 9).

Recent studies strongly support the role of neuroinflammation in PD [18]. The CNS innate immune surveillance is primarily mediated by microglia. Once activated by immunological challenges and neuronal injuries, microglia are recognized to induce neuronal damage [19]. It is interesting to note that TSG exerted neuroprotection against DA neurotoxicity induced by LPS and MPP^+^, two toxins with different modes of functions. LPS directly led to microglial activation and then caused DA neuronal damage. On the contrary, MPP^+^ directly resulted in DA neuronal death and subsequently the injured DA neurons released kinds of neurotoxic soluble factors, such as α-synuclein and damage-associated molecular patterns (DAMPs), which in turn induced microglial activation. These reactivated microglia again produced pro-inflammatory factors and contributed to continuous DA neuronal damage [20]. Either kind of microglial activation induced by LPS and MPP^+^ would further cause DA neuronal death in a “self-propelling” vicious cycle and consequently induce the progressive DA neurodegeneration. In this study, as shown in Fig. 2, TSG-mediated neuroprotection was detected in neuron-microglia cultures but not in neuron-enriched cultures, suggesting microglia were at least required for TSG-mediated DA neuroprotection.

Furtherly, several lines of evidence have confirmed that microglial activation and the subsequent release of pro-inflammatory and cytotoxic factors, such as NO, TNFα, IL-1β, and PGE_2_, contribute to PD pathogenesis. Analysis from postmortem brains demonstrates that microglial activation and elevated pro-inflammatory factors, such as TNFα, IL-1β, and NO, have been discerned in patient SN [21]. Therefore, inhibition of microglia-induced neuroinflammation might possess a potential therapeutic strategy for PD. Consistent with our previous findings that TSG decreased LPS-elicited pro-inflammatory factors release in BV2 cells [22], we here found TSG reduced LPS-induced pro-inflammatory factors production in neuron-glia co-cultures. These findings implied that inhibition of microglial activation and the subsequent pro-inflammatory factors production was involved in TSG-produced neuroprotection on DA neurons.

Among the pro-inflammatory and neurotoxic factors released by activated microglia, ROS, such as superoxide, play a pivotal role in DA neuronal death [23]. Also, superoxide reacts with NO to form highly toxic intermediates, such as peroxynitrite, to significantly contribute to DA neurodegeneration. Thus, inhibition of microglial ROS production, rather than the other pro-inflammatory factors, was the most effective in DA neuron protection [24, 25]. Furthermore, one of the major sources of ROS is NADPH oxidase. The inhibition and the genetic deletion of NADPH oxidase could evoke neuroprotection against LPS, paraquat, and rotenone-induced DA neurodegeneration [26, 27]. In addition, an increasing body of evidence indicates intracellular ROS induce the activation of MAPK and NF-κB signaling cascades in the activated microglia [28, 29]. The ROS-scavenging enzyme catalase and the inhibitor of NADPH oxidase activity significantly inhibited MAPK and NF-κB signaling activation, thereby linking NADPH oxidase-produced ROS with inflammation-related MAPK and NF-κB transcriptional activation [30, 31]. Here, this study implied that TSG would ameliorate NADPH oxidase and MAPK and NF-κB cascade activation and finally decrease pro-inflammatory factors production.

Besides, TSG exerted DA neuroprotection in neuron-astroglia cultures in addition to neuron-microglia cultures. Astroglia, the most abundant CNS non-neuronal cell population, are well conceptualized as an inert scaffold or housekeeping cells. Also, astroglia could release neurotrophic factors, such as BDNF and GDNF, and further promote neuronal survival and maintain synaptic homeostasis [32]. Thus, astroglia usually confer neuroprotection and attenuate early symptoms of neurodegenerative diseases. Furthermore, astroglia are the active participants in propagating and modulating neuroinflammation [33]. Astroglia become activated by inflammatory cytokines, such as IL-1β, and secrete several neurotoxic substances along with an enhanced GFAP protein expression. This enhanced GFAP expression relates to the astroglia activation severity [34]. However, strong activation of astroglia results in the secretion of a large number of chemokines, cytokines, ROS, and pro-inflammatory factors, further affecting the cellular state of surrounding cells, such as neurons, microglia, and astroglia themselves, thereby leading to excitotoxicity and neurodegeneration [35]. In this study, TSG had no significant effects on astroglial activation. We speculated this phenomenon was because such low dose of LPS used in this study mainly induced microglial activation (but not astroglial activation) to further caused DA neuronal loss. However, the role of astroglia on the low inflammation-induced DA neurodegeneration requires further illumination.

Moreover, TSG induced neurotrophic factors release in neuron-glia and neuron-astroglia cultures. The neutralization of astroglial neurotrophic factors weakened TSG-mediated neuroprotection. These findings suggested astroglia-derived neurotrophic effects were involved in TSG-produced neuroprotection. A battery of studies has confirmed that the neurotrophic factors are indispensable for the maintenance in the developing and adult brain [36]. Lack of neurotrophic factors led to the neuronal loss and the progression of PD [37]. However, studies from clinical trials demonstrated, as a clinical agent, BDNF was problematic with minimal blood-brain barrier (BBB) penetrability, short serum half-life, poor bioavailability, and limited diffusion in CNS tissues [38]. As a macromolecule, GDNF could not pass the BBB either although direct infusion of GDNF into the brain is eventually required to aim at the therapeutic purpose [39]. Meanwhile, GDNF protein infusion into brain was quite difficult to develop into a long-term therapeutic approach [40]. In this regard, there has been strong interest on developing small molecular mimetics of neurotrophic factors or treatment drugs to enhance endogenous neurotrophic factors production and consequently possess the therapeutic potential.

Interestingly, this study found that TSG alone increased DA uptake and DA neuronal number and neurotrophic factors release. It has been confirmed that BDNF could mediate neuronal survival, differentiation, synaptic plasticity, and neurogenesis [41]. Recent studies demonstrate that post-ischemia treatment with exogenous BDNF not only exerts neuroprotection but also induces neuronal regeneration [42]. Here, we speculated that TSG seemed to not only protect DA neurons but also elicit DA neurogenesis. However, attempts to elucidate this phenomenon have become an area of great interest in future.

At present, PD therapeutic interventions are focused on the symptom control and fail to halt the progressive neurodegenerative process. The efficacy decline of the dopamine precursor l-dihydroxyphenylalanine (l-DOPA), one of the gold-standard drugs used for PD treatment, and the occurrence of severe side-effects following its long-term use highlight a need to develop more effective therapeutic strategies [43]. Current observations indicate that inhibition of microglia-mediated neuroinflammation could attenuate DA neuronal loss in various PD models. The clinical trials concerning the therapeutic actions of anti-inflammatory drugs on the natural progression of PD is too limited to draw any further conclusion, although a great deal of encouraging experimental evidence is shown [3]. However, the low success rate of translating potential anti-inflammatory drugs from animal studies to patients was exposed. Thus, an urgent need for better approaches to anti-inflammatory drug design was prompted. Recent studies confirm that TSG could go through BBB [44]. Consequently, TSG presents numerous biological functions in neurological disorder treatments. In sleep-deprived mice, amyloid-β (Aβ)-injected aging mice, and kainic acid-induced brain damage mice, TSG restored memory impairment through the induced expressions of erythropoietin, PPAR-γ coactivator 1α (PGC-1α), and hemoglobin in astroglia and neurons of the hippocampus [45]. Furthermore, TSG apparently reduced the binding of NF-*κ*B to its DNA element in the iNOS promoter and thus inhibited neuroinflammation [46]. In Aβ-elicited rat AD model, TSG reversed the increased amyloid precursor protein (APP) expression and the downregulation of Src and NR2B mRNA and protein levels and finally improved the cognitive impairment [47]. In ischemia reperfusion-injured rat model, TSG attenuated animal behavior changes and neurological function scores via increasing the expressions of NGF, growth-associated protein 43, and PKA catalytic subunit proteins [48]. Here, the present study further revealed that TSG not only inhibited microglia-mediated neuroinflammation but also enhanced astroglia-derived neurotrophic effects and combination of these two beneficial effects proved to protect DA neurons against LPS-induced neurotoxicity. Despite this apparently perspective for TSG potential treatment for PD, most of the findings resulted from acute neuroinflammation-induced DA neuronal damage. However, the chronic inflammation could be one of the most important contributors to the pathogenesis of PD. Furtherly, the role of microglia and astroglia responding to chronic inflammation might be different from those to acute inflammation. The underlying mechanisms warrant further rigorous investigation.

## Conclusions

This study demonstrates TSG-protected DA neurons against LPS-induced neurotoxicity through dual modulation on glial cells by attenuating microglia-mediated neuroinflammation and enhancing astroglia-derived neurotrophic effects. These findings might open new alternative avenues for PD treatment.

## Abbreviations

BCA: Bicinchoninic acid
BDNF: Brain-derived neurotrophic factor
DA: Dopamine
DOPAC: 3, 4-Dihydroxyphenylacetic acid
EDTA: Ethylenediaminetetraacetic acid
GDNF: Glial cell line-derived neurotrophic factor
GFAP: Glial fibrillary acidic protein
HVA: Homovanillic acid
Iba-1: Ionized calcium-binding adapter molecule-1
IL-1β: Interleukin-1 beta
LPS: Lipopolysaccharide
MPTP: 1-Methyl-4-phenyl-1,2,3,6-tetrahydropyridine
NO: Nitric oxide
PBS: Phosphate-buffered saline
PD: Parkinson’s disease
PGE_2_: Prostaglandin E_2_
ROS: Reactive oxygen species
SN: Substantia nigra
TH: Tyrosine hydroxylase
TNFα: Tumor necrosis factor-alpha
TSG: Tetrahydroxystilbene glucoside
WST-1: Water-soluble tetrazolium salt
